# Off-Lattice Monte-Carlo Approach for Studying Nucleation and Evaporation Phenomena at the Molecular Scale

**DOI:** 10.3390/ma14092092

**Published:** 2021-04-21

**Authors:** Panagiotis E. Theodorakis, Yongjie Wang, Aiqiang Chen, Bin Liu

**Affiliations:** 1 Institute of Physics, Polish Academy of Sciences, Al. Lotników 32/46, 02-668 Warsaw, Poland; wang@ifpan.edu.pl; 2 Tianjin Key Laboratory of Refrigeration Technology, Tianjin University of Commerce, Tianjin 300134, China; chenaiqiang@tjcu.edu.cn

**Keywords:** droplet, Monte-Carlo simulation, off-lattice models, nucleation, evaporation

## Abstract

Droplet nucleation and evaporation are ubiquitous in nature and many technological applications, such as phase-change cooling and boiling heat transfer. So far, the description of these phenomena at the molecular scale has posed challenges for modelling with most of the models being implemented on a lattice. Here, we propose an off-lattice Monte-Carlo approach combined with a grid that can be used for the investigation of droplet formation and evaporation. We provide the details of the model, its implementation as Python code, and results illustrating its dependence on various parameters. The method can be easily extended for any force-field (e.g., coarse-grained, all-atom models, and external fields, such as gravity and electric field). Thus, we anticipate that the proposed model will offer opportunities for a wide range of studies in various research areas involving droplet formation and evaporation and will also form the basis for further method developments for the molecular modelling of such phenomena.

## 1. Introduction

Nucleation and evaporation of liquid droplets [1,2,3,4,5] in contact with solid substrates are ubiquitous in nature (e.g., condensation and evaporation of rain droplets) and many technological applications, such as phase-change cooling [6] and boiling heat transfer [7]. These phenomena are complex and they can be influenced by different factors, such as thermodynamic conditions [8], substrate properties [9], and impurities [10]. Even when one considers the simplest case of droplets without impurities onto unstructured and smooth substrates under constant thermodynamic conditions, the understanding of these processes continues to pose challenges. The origin of these phenomena lies in the interactions among the molecules of the system, which is difficult to capture with experimental or theoretical methods and continuum simulations. Certain challenges also exist in the case of molecular modeling, as these phenomena are non-equilibrium processes that require the exchange of molecules between the liquid drop and the surrounding environment. To this end, various molecular models have been proposed over the last years, which have aimed at overcoming those challenges and have also led to relevant investigations of these fascinating phenomena [11,12,13,14,15,16,17,18,19,20,21,22,23]. For example, molecular-level studies have focused on evaporating droplets with nanoparticles. In this case, as the liquid evaporates, various patterns form, which, in turn, can be compared with experimental observations [11,12,18,24]. Despite these studies, a more accurate description of nucleation and evaporation phenomena requires the development of new simulation frameworks, even for simple systems, for example, single-component systems without the presence of external fields.

Fillipponi and Giammatteo have investigated the classical nucleation process by using kinetic Monte-Carlo simulation (KMC) [23]. Their approach was applied for a wide range of temperatures providing descriptions in line with the classical nucleation theory, in particular with respect to parameters describing the average population distribution of the nuclei size. However, this approach is stochastic in nature, requiring an approximation to the exact dynamics by generating a set of random integer numbers from Poisson distributions, which is also computationally demanding. A similar approach has been employed to study the nucleation-growth processes of transition metal dichalcogenides [16]. Reducing the volume of a system or using schemes based on the grand canonical ensemble one could simulate the droplet formation [1]. In the latter approach, however, the formation is spontaneous, which limits a detailed investigation of the droplet nucleation mechanisms.

Various molecular models have been proposed to investigate droplet evaporation processes. Zhang et al. [14] have employed molecular dynamics simulations of all-atom force-fields to investigate the wetting and evaporation of salt-water nanodroplets on platinum surfaces focusing on the patterns formed as a result of evaporation. Although this method offers an accurate atomistic description of the system, it is computationally demanding and requires particular care for carrying out the simulations and their subsequent analysis. In contrast, other molecular models, such as those based on Monte-Carlo (MC) techniques, can overcome such limitations [12,15,18,19]. In particular, MC models offer flexibility in choosing the various moves that would transform the system from one state to the next (including attempts to remove or add particles) and confirming the acceptance of such moves by using the Metropolis criterion. For example, based on the latter approach, Rabani et al. have created a two-dimensional (2D) [18] and a three-dimensional (3D) [19] model for a liquid droplet laden with nanoparticles, which has later been applied in further applications, such as the study of instabilities in dewetting nanofluids [20,21] and patterns obtained from drying colloidal nanoparticle solutions [22]. A more recent version of the 2D version of the Rabani model [18] considers a chemical potential that depends on time and the radius of the droplet. In this study, Zhang et al. have found different drying patterns that are in good agreement with experimental results [12]. Finally, a similar MC approach on the lattice and the link to hydrodynamics has been discussed in a recent study by Areshi et al. [15]. A common feature of all the above MC studies is the use of lattice models, which considerably simplifies the implementation of the applied method and overcomes various difficulties in dealing with the exchange of particles at the liquid–vapor interface between the droplet and the surrounding vapor. However, if we would like to better capture the dynamic behavior of the nucleation and the evaporation of a droplet, an off-lattice approach would be desirable along with a more natural representation of the system (flexibility in choosing the force-field), especially close to the droplet surface where these phenomena manifest.

Here, we address these issues by proposing an off-lattice MC approach for studying droplet nucleation and evaporation phenomena. The approach is based on a standard off-lattice MC scheme in the canonical ensemble for the bulk of the droplet, which is additionally equipped with the ability of removing and adding particles at the liquid–vapor (LV) interface by using a suitable grid and the chemical potential. Here, the implementation of the model is illustrated in a simple system of Lennard-Jones (LJ) particles, but it can easily be extended for systems that include nanoparticles or other molecules. Moreover, the developed method can be used with any available forcefield, be it all-atom or coarse-grained. Hence, we anticipate that our approach will form the basis for further conceptual developments in this area. In the following, we discuss the method details and provide a parametric study of the proposed model. An implementation of the model as Python code is available as Supplementary Information.

## 2. Simulation Model and Method

Our system consists of an implicit substrate and a droplet of coarse-grained beads that interact by means of the LJ 12–6 potential:

(1) $$U^{12-6}\left(r\right)=4\varepsilon_{d}\left[{\left(\frac{\sigma_{d}}{r}\right)}^{12}-{\left(\frac{\sigma_{d}}{r}\right)}^{6}\right].$$

Here, only interactions between beads at distances, *r*, smaller than a cutoff distance are considered. This cutoff is $r_{c}=2.5\sigma$, where σ is the unit of length. The LJ potential is also shifted at the cutoff. As a result the energy $U^{12-6}\left(r_{c}\right)=0$. The parameter $\varepsilon_{d}$ tunes the strength of the LJ interaction between the beads and is measured in units of ε. Here, we keep $\varepsilon_{d}=\varepsilon$ and the Boltzmann constant, $k_{B}$ is taken as unity. The interaction between the droplet beads and the substrate is realized through an LJ 9–3 potential, where the exponents result from the integration of the LJ 12–6 potential over the substrate [25,26,27,28]. Hence, the substrate is only implicitly present in the system representing a smooth and unstructured substrate of `infinite’ thickness. The LJ 9–3 potential, $U^{9-3}\left(r\right)$, between each bead and the substrate reads

(2) $$U^{9-3}\left(z\right)=4\varepsilon_{s}\left[{\left(\frac{\sigma_{s}}{z}\right)}^{9}-{\left(\frac{\sigma_{s}}{z}\right)}^{3}\right],$$

where the parameter $\varepsilon_{s}$ is used to vary the interaction between the substrate and the droplet beads, while $\sigma_{s}=\sigma_{d}$=σ for simplicity. The distance, *z*, is simply the distance between the substrate and the droplet beads in the direction that is normal to the substrate (*z* direction). As in the case of the LJ 12–6 potential, the LJ 9–3 potential is cut and shifted at the same cutoff distance, i.e., $z_{c}=2.5\sigma$.

**Figure 1:**
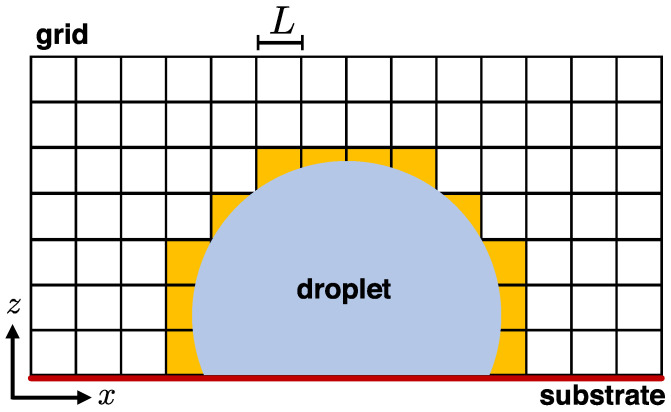
Schematic diagram of the system setup in two dimensions (2D) for the sake of simplicity and clarity. The grid has a size *L*. The cells at the liquid–vapour interface that are relevant for the addition and the removal of particles are shaded with yellow color.

The simulation approach of this study is designed to harvest the advantages of off-lattice MC methods and the same time be suitable for investigating nucleation and evaporation phenomena at the molecular scale. The bulk of the droplet is simulated by using the standard NVT MC simulation method, but the interface of the droplet is continuously tracked and treated differently. In particular, a three-dimensional (3D) grid is created, which is used to identify the liquid–vapour (LV) interface of the droplet by tracking the density of the beads in the grid cells. A similar concept has been used in the case of the volume of fluid method [29]. However, in our case this is only applied to identify the LV interface of the droplet (Figure 1). Then, beads belonging to the interface are treated with an MC approach based on a Hamiltonian that involves the chemical potential, as, for example, in the case of the Rabani et al. model [18]. Our approach can be readily extended to incorporate other concepts, such as that of a varying chemical potential depending on the droplet radius and time, as in the case of the Zhang et al. model [12]. In addition, the model can be used with different force-fields (including, also, all-atom force-fields), which renders it particularly suitable for multicomponent systems (e.g., liquid droplets with nanoparticles). External fields (e.g., electric field, gravity, etc.) can easily be added to the model as additional energy terms. While the method is described for MC simulations in this study, possible extensions based on the molecular dynamics (MD) method are conceivable [30,31,32].

A cubic grid of mesh size $L>r_{c}$ is initially created across the whole simulation domain with periodic boundary conditions applied in all directions. In the following, L=4σ. Each cubic cell of the grid is assigned beads according to their positions and the density of the cells is calculated. Cells with a density below a certain threshold, $\rho_{c}$ (e.g., below the bulk density of the droplet), will be identified as cells that contain particles belonging to the LV interface. While the use of the grid facilitates the identification of the droplet interface, it also provides an efficient way of finding the neighboring particles during the calculation of the system energy by searching only the neighboring cells. In our case, this is implemented by using for each cell a Python dictionary that holds its neighboring cells. The interaction energy of the system is described by the following Hamiltonian,

(3) $$H=\sum_{<\mathrm{ij}>}U_{\mathrm{ij}}^{12-6}\left(r\right)+\sum_{<i>}U_{i}^{9-3}\left(z\right)-\mu N,$$

where <ij> indicates interactions between all pairs of atoms that are found at distances, *r*, smaller than the cutoff, $r_{c}$. Similarly, the sum over each bead <i> refers to the interaction of each bead with the substrate, when the distance between the bead and the substrate is smaller than the cutoff, $z_{c}$. *N* is the number of particles in the system and μ is the chemical potential, which is the energy cost when adding or removing a particle to the system. This is a property of the particular component, which implies that the simulation of multicomponent systems would require the definition of the chemical potential for each particle type. In practice, the chemical potential here reflects the tendency for evaporation or nucleation and is measured in units of ε. In particular, more negative μ values would favor evaporation, while increasing μ would favor nucleation. The generation of subsequent states of the system is based on the realization of local MC moves and the addition or removal of beads to realize the evaporation and nucleation phenomena at the droplet interface. The new state of the system is accepted by using the Metropolis probability, $p_{\mathrm{acc}}=\min\left[1,\exp\left(-\Delta H/K_{B}T\right)\right]$, where ΔH is the energy difference between a new attempted state and the current state of the system. In the following, we discuss in detail how this framework is specifically implemented in the nucleation and evaporation cases.

### 2.1. Nucleation

After the initialization of the grid and the assignment of each particle to the respective grid cell, the system advances to subsequent states by randomly choosing a particle from the droplet and realizing local MC particle moves. As usual, the number of such attempts corresponds to the number of particles in the system. The decision of accepting the new state of the system is based on the Metropolis criterion. Then, an attempt to add a new particle to the system takes place. A particle is randomly chosen and its cell and neighboring cells are identified. An attempt to add a new particle in these cells takes place. If the new particle is placed at a distance $r_{n}$ ($\sigma <r_{n}<r_{c}$), then the new state is accepted according to the Metropolis criterion by considering the Hamiltonian of Equation (3) and the MC cycle is completed. In this approach, one can tune the distance threshold, $r_{n}$, the ratio between the attempts of local moves and adding new particles, the chemical potential, μ, the temperature of the system, *T*, and the size of the grid cells, *L*. The detailed-balance condition would require an equal probability to remove a particle from the system. However, one may consider in our case that this probability is incorporated in the choice of the chemical potential, μ, of the system. Moreover, the chosen simulation protocol would speed up the study of the droplet nucleation in this case, which is itself a non-equilibrium process for the system. In the following, the protocol for evaporation can be used as well for studying nucleation phenomena by increasing the value of the chemical potential, in this way favoring the addition of particles to the system.

### 2.2. Evaporation

The grid is initialized and particles are assigned to cells according to their positions similar to what is done in the case of nucleation. Apart from the standard local moves for all system beads, additional attempts to add and remove a particle take place in each MC cycle for cells being at the LV interface. In particular, a bead is randomly selected and its cell is identified. If the density of the cell is below a density threshold, $\rho_{c}$, this cell contains beads of the LV interface. Then, a standard local move is attempted. If the new position of the bead is within a threshold distance, $r_{n}$, ($\sigma <r_{n}<r_{c}$) from its neighbours, the new position of the bead is accepted according to the Metropolis criterion. If the distance between the particle and all its neighbors is larger than $r_{n}$, the selected bead is removed according to the Metropolis probability and considering the Hamiltonian of Equation (3). In the latter case, particles that have moved far from the droplet according to the distance criterion, $r_{n}$, are considered as evaporated particles. From the cell of the previously-selected particle, we randomly choose a bead and attempt to remove it. This attempt is accepted according to the Hamiltonian of Equation (3) and the Metropolis criterion. Finally, an attempt to add a new bead in the selected cell takes place. The addition of the new particle is also accepted according to the Hamiltonian of Equation (3) and the Metropolis criterion.

The above simulation protocols take advantage of the flexibility of the MC approach in adding and removing particles from the system, as well as the use of the grid cells. These protocols constitute the basis upon which further methodology developments could be proposed in this area in the future, including methods based on MD or even multiscale protocols [33]. Therefore, various modifications of the algorithms are expected for improving and adjusting the model to the particular problem at hand. For this reason and for better understanding the behavior and limits of the method, we provide the implementation of our approach as a Python code. In the following, we present results from our simulations that illustrate the impact of the various parameters on the model.

## 3. Results

We have performed a broad exploration of the model parameters, *T*, $\varepsilon_{s}$, $r_{n}$, $\rho_{c}$, and μ. In particular, we have considered the following range of values for each parameter: $0.2\varepsilon /k_{B}\leq T\leq 1.0\varepsilon /k_{B}$, $1.1\sigma \leq r_{n}\leq 1.5\sigma$, $0.6\sigma^{-3}\leq \rho_{c}\leq 0.9\sigma^{-3}$, and −4.0ε≤μ≤2.0ε. Of course, the choice of the system temperature, *T*, affects proportionally all the related energy parameters of the model. Based on our analysis, we have found that the choice of $r_{n}$ plays an important role in the model. The influence of the latter and all parameters of the model will be discussed in more detail in the following.

**Figure 2:**
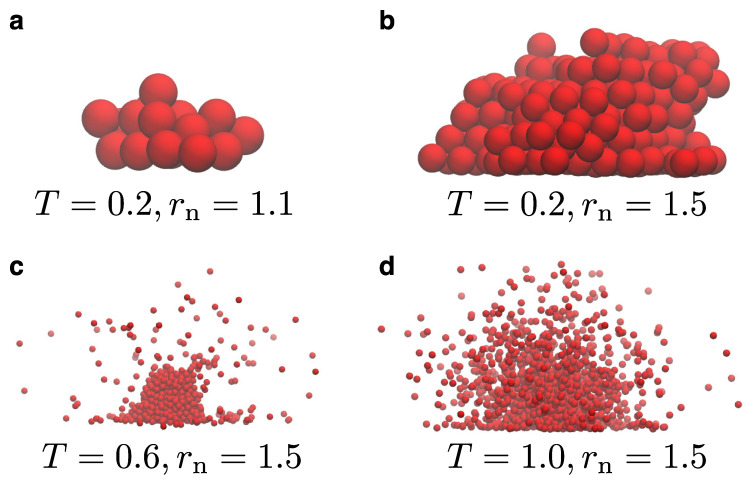
(**a**–**d**) Snapshots obtained from the nucleation algorithm for various temperatures, *T* (in units of $\varepsilon /k_{B}$) and $r_{n}$ (in units of σ), as indicated. For all cases, μ=−1.0ε, $\varepsilon_{s}=1.5\varepsilon$, and $\rho_{c}=0.7\sigma^{-3}$. Snapshots are taken after the realisation of $10^{4}$ MCS. The scale of each snapshot has been adjusted in order to make the snapshots visually clearer.

**Figure 3:**
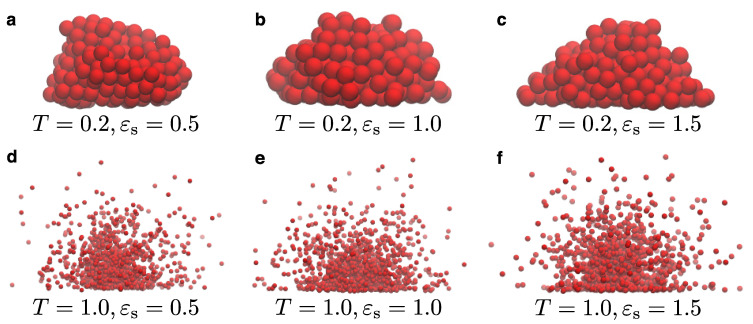
(**a**–**f**) Snapshots obtained from the nucleation algorithm for various temperatures (in units of $\varepsilon /k_{B}$) and droplet–substrate attraction strength $\varepsilon_{s}$ (in units of ε), as indicated. For all cases, μ=−1.0ε, $r_{n}=1.4\sigma$, and $\rho_{c}=0.7\sigma^{-3}$. The scale of each snapshot has been adjusted in order to make the snapshots visually clearer.

Figure 2 illustrates representative snapshots for the model for various choices for the temperature and the parameter $r_{n}$. We remind the reader that $r_{n}$ is used to distinguish whether a particle at the LV interface belongs to the liquid droplet or is part of the vapor. This value is larger than the size of the droplet beads, $\sigma_{d}$, and up to 1.5σ in our case, which is a distance within the first and second interaction shells of particles in the bulk. Our simulations start by initially placing a single particle onto the substrate, whence the droplet starts to grow. As the droplet grows, we observe that vapor particles are absent at lower temperatures (e.g., $T=0.2\varepsilon /k_{B}$), or their presence is negligible during the simulation. Moreover, the addition of new particles to the droplet takes place faster when $r_{n}$ is larger. In general, we have found that values of $r_{n}$ larger than 1.1 are required to start the nucleation process at larger temperatures, when the rest of the model parameters are kept the same. As the temperature of the system increases, we also observe the presence of vapor around the liquid droplet. Hence, in this sense the model works as expected by only preserving the vapor close to the LV interface. Moreover, we can clearly distinguish the boundaries of the LV interface. As the temperature further increases, thermal fluctuations become more pronounced in the system both at the bulk and the LV interface. In all cases, vapor exists only close to the LV interface and particles far away from the interface will eventually be removed during the simulation. As a result, the computational time of the simulation spent on vapor particles in the case of our model is rather small. In the snapshots of Figure 2, we can also see the impact of the substrate potential, which is rather large in these particular cases, namely $\varepsilon_{s}=1.5\varepsilon$. More specifically, we can observe the distortion of the droplet contact-line at higher temperatures with beads lying nearby onto the substrate. The influence of the substrate can be visually summarized by the snapshots of Figure 3 for the lowest ($T=0.2\varepsilon /k_{B}$) and the highest ($T=1.0\varepsilon /k_{B}$) temperatures considered in our study. We found that the strength of the substrate potential has a small effect on the growth rate of the droplet, independently of the temperature. However, the final configurations will be different under the influence of the substrate potential, especially close to the contact line. For example, we can observe the formation of a precursor layer at the contact line of the droplet at higher temperatures. Moreover, smaller contact lines are observed for the droplet for both temperatures, when the strength of the substrate potential is larger.

**Figure 4:**
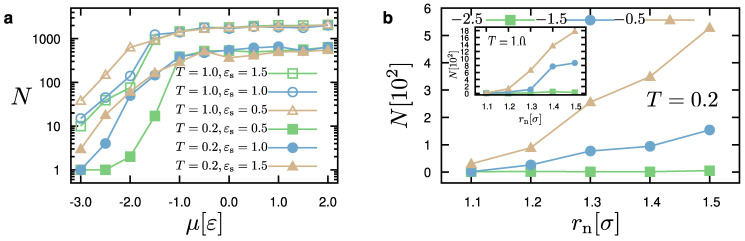
(**a**) Dependence of the number of particles, *N*, on the chemical potential, μ, for different temperature, *T* (in units of $\varepsilon /k_{B}$) and attraction strength, $\varepsilon_{s}$ (in units of ε), as indicated. $r_{n}=1.5\sigma$ and $\rho_{c}=0.7\sigma^{-3}$; (**b**) Dependence of the number of particles, *N*, in the system as a function of the parameter $r_{n}$ for different cases of the chemical potential, μ=−0.5,−1.5, and −2.5ε, as indicated. Main panel shows data for temperature, $T=0.2\varepsilon /k_{B}$, while inset presents data for $T=1.0\varepsilon /k_{B}$. For all cases, $\rho_{c}=0.9\sigma^{-3}$, $\varepsilon_{s}=1.5\varepsilon$.

Figure 4 presents results for the dependence of the number of particles in the system as a function of the chemical potential, μ, and the distance parameter, $r_{n}$. As mentioned before, these parameters significantly affect the behavior of the system. We can observe that the number of particles increases as a function of the chemical potential during a simulation of $10^{4}$ MCS. Above a certain value, namely μ=−1.0ε, the influence of the chemical potential is small, independently of the temperature of the system. Moreover, we observe that the number of particles, *N*, is rather larger in the case of a smaller substrate–droplet interaction, that is $\varepsilon_{s}=0.5\varepsilon$. A similar behavior is observed in the case of a higher temperature ($T=1.0\varepsilon /k_{B}$). The dependence of the number of particles of the system, *N*, on $r_{n}$ is also significant. Within the time of the simulation ($10^{4}$ MCS), we can see that small values of $r_{n}$ restrict the addition of new particles to the droplet. As $r_{n}$ becomes larger, we observe a greater ability of adding particles to the system. This ability also depends on the value of the chemical potential. In particular, the higher the chemical potential, the higher is the dependence on $r_{n}$. By examining the structures of all our cases, we have observed that the value of $r_{n}$ affects the rate of droplet growth, but it generally does not influence the droplet configurations, for $r_{n}>1.1$σ. We have also seen that $r_{n}=1.1$σ rather hinders the growth of the droplet on the substrate and the formation of droplets has not been possible for various choices of model parameters within the available time of the simulation when the temperature increases. The above discussion is consistent throughout the extensive parameter exploration considered in this study.

**Figure 5:**
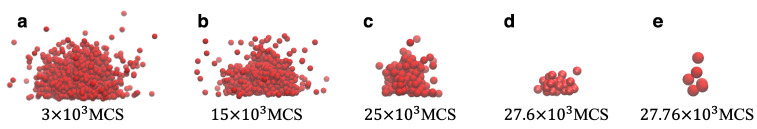
(**a**–**e**) Various snapshots of droplet evaporation at different times (measured in MCS), as indicated. Here, $T=1.0\varepsilon /k_{B}$, μ=−1.0ε, $\varepsilon_{s}=1.0\varepsilon$, $r_{n}=1.5\sigma$, and $\rho_{c}=0.7\sigma^{-3}$. The scale of each snapshot has been adjusted in order to make the snapshots visually clearer.

We now turn our discussion to the evaporation model, which is the main focus of our work. Figure 5 presents various snapshots during the evaporation process of a droplet for a particular case. The initial configuration of the system is a droplet that contained 1578 particles and was created with our nucleation algorithm. At each stage of the evaporation process, we can clearly distinguish the bulk of the droplet and the surrounding vapor in the system. During evaporation, the droplet changes configurations by using local MC moves and exchanging particles at the LV with the surrounding vapor phase. The algorithm produces consistent results independently of the droplet size and until the droplet has fully evaporated.

**Figure 6:**
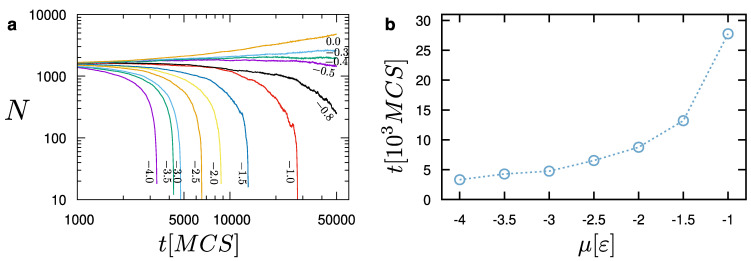
(**a**) Dependence of the number of particles during evaporation for different values of the chemical potential, μ (−4.0≤μ≤0.0) in units of ε, as indicated, as a function of time measured in the number of Monte-Carlo steps (MCS). The liquid (droplet) and the vapour phases are in equilibrium for μ=−0.4ε; (**b**) Time (measured in MCS) required for the full evaporation of the droplet, as a function of the chemical potential, μ. $T=1.0\varepsilon /k_{B}$, $r_{n}=1.5\sigma$, $\rho_{c}=0.7\sigma^{-3}$, and $\varepsilon_{s}=1.0\varepsilon$.

The rate of evaporation depends on the choice of the chemical potential. As shown in Figure 6, more negative values of the chemical potential lead to faster evaporation. In contrast, values larger than μ=−0.4ε will lead to the addition of particles to the droplet. For the particular choice of parameters, we observe an equilibrium between the liquid and the surrounding vapor particles for μ=−0.4ε. Establishing such an equilibrium is a key element for the success of our model. This indicates that the liquid droplet can be reliably simulated while coexisting with the surrounding vapor particles. In all evaporation cases (μ<−0.4ε), we have found that the droplet initially evaporates at a slower pace. When the droplet size reaches about 250 particles in this case, then its evaporation accelerates (Figure 6). Hence, we can distinguish two different behaviors, which are determined by the size of the droplet. By examining the snapshots of the system at each evaporation stage (e.g., see Figure 5), we have found that the pace of evaporation is only affected by the chemical potential for a given set of model parameters. The model produces consistent results and configuration changes take place as expected. As the chemical potential increases, droplet evaporation requires larger times, which grow exponentially as the chemical potential reaches the point that the liquid and vapor particles are in dynamic equilibrium (Figure 6b).

**Figure 7:**
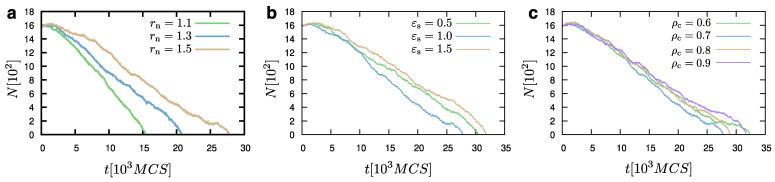
Evaporation of a droplet at temperature $T=1.0\varepsilon /k_{B}$, expressed through the number of beads, *N*, of the system as a function of time, *t*, for different values of $r_{n}$ (**a**), $\varepsilon_{s}$ (**b**), and $\rho_{c}$ (**c**), as indicated on each graph. μ=−1.0ε, $\rho_{c}=0.7\sigma^{-3}$, and $\varepsilon_{s}=1.0\varepsilon$. $r_{n}=1.5\sigma$ in panels (**b**,**c**).

Our evaporation model is not sensitive to the choice of the parameter $\rho_{c}$ (Figure 7), as it has also been found in the case of the nucleation protocol. In addition, the influence of the wall potential has a small effect when $\varepsilon_{s}=0.5$ and 1.0ε, while the case $\varepsilon_{s}=1.5\varepsilon$ would lead to a slight delay of the complete droplet evaporation, due to the extra energy that the substrate provides to the particles. However, $r_{n}$ would significantly affect the evaporation process. In particular, smaller values of $r_{n}$ (e.g., $r_{n}=1.1\sigma$) would lead to a faster evaporation of the droplet, since beads at a distance $r_{n}=1.1\sigma$ away from the LV will be already considered as part of the vapour. In contrast, larger values of $r_{n}$ (e.g., $r_{n}=1.5\sigma$, which is also a more natural choice as it includes the first shell of neighbours), would lead to larger times for the complete evaporation of the droplet. Hence, as in the case of nucleation, the choice of $r_{n}$ is crucial in the case of the evaporation algorithm.

**Figure 8:**
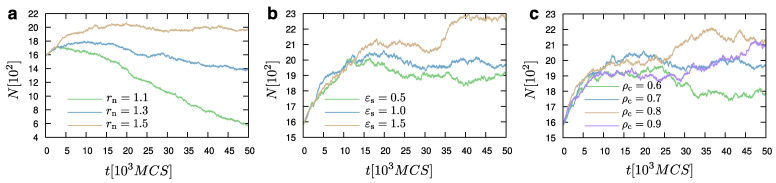
Same as in Figure 6, but μ=−0.4ε. An equilibrium between the liquid droplet and the surrounding vapour exists for $r_{n}=1.5\sigma$. $r_{n}=1.5\sigma$ in panels (**b**,**c**), while cases with different $r_{n}$ are shown in panel (**a**), as indicated.

**Figure 9:**
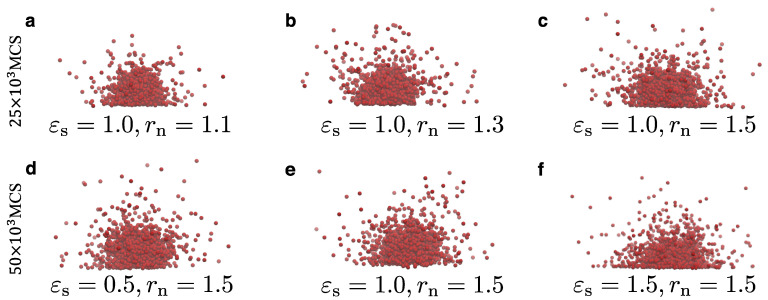
(**a**–**f**) Snapshots of the system at $T=\varepsilon /k_{B}$ for different values of $\varepsilon_{s}$ and $r_{n}$ at different times (top panels: $25\times 10^{3}$ MCS; bottom panels: $50\times 10^{3}$ MCS). For all cases, μ=−0.4ε and $\rho_{c}=0.7\sigma^{-3}$. The scale of each snapshot has been adjusted in order to make the snapshots visually clearer.

The choice of $r_{n}$ can affect the dynamic equilibrium of the system. Hence, once $r_{n}$ is chosen, it has to remain the same throughout a study. For example, when μ=−0.4ε and $r_{n}=1.5\sigma$, we observe the dynamic equilibrium between the liquid and the vapor phases (Figure 8). However, when another value of $r_{n}$ (e.g., $r_{n}=1.2\sigma$) is chosen, the value of the chemical potential required to establish the equilibrium between the droplet and its surrounding vapor particles, would also change. Figure 9 illustrates characteristic snapshots at time $25\times 10^{3}$ MCS for different $r_{n}$ cases. As previously mentioned, the evaporation process is faster in the case of smaller $r_{n}$, which would lead to a smaller size of the droplets. Comparing the configurations during evaporation for the different cases of $r_{n}$, we do not see any significant structural differences. Hence, the dynamic equilibrium between the liquid and the vapor phases can be obtained again by a proper choice of the chemical potential, μ. For the case that a dynamic equilibrium is established for $r_{n}=1.5\sigma$, we have included a movie as Supplementary Information. We have also found that changes in $\rho_{c}$ or $\varepsilon_{s}$ would still maintain the equilibrium in the system for the same value of the chemical potential (Figure 8). Moreover, different choices for $\varepsilon_{s}$ may slightly shift this equilibrium to larger or smaller droplets without the requirement to change the value of the chemical potential. In particular, we observe that larger values of $\varepsilon_{s}$ would favour a larger droplet size (Figure 8). The effect of the substrate attraction strength on the droplet shape for droplets in dynamic equilibrium between the vapour and the liquid phases, in particular at the contact line, can be seen in the snapshots of Figure 9.

## 4. Conclusions

In this study, we have proposed an off-lattice approach, which can be used to simulate nucleation and evaporation phenomena of droplets at the molecular scale. The model can be easily extended for any forcefield, be it atomistic or coarse-grained. We have taken advantage of the flexibility of the MC approach combined with a 3D grid. The grid is used to identify the position of the LV interface, where the addition and removal of particles take place. Moreover, vapor particles far from the LV interface are naturally removed by the algorithm during the simulation, which also makes our approach computationally efficient. The model works as we expected with the chemical potential controlling the processes and nicely capturing the liquid droplet as well as the vapor particles around the droplet. Moreover, our evaporation protocol is able to establish a dynamic equilibrium between the droplet and the surrounding vapor particles. Hence, evaporation, a dynamic equilibrium between liquid and vapor, as well as nucleation phenomena can be modeled based on this approach. By examining a broad range of values for the model parameters, we have found that the parameter $r_{n}$ should be chosen carefully and remain constant during the study of a particular problem at hand. Further improvements of the model are conceivable given the flexibility of the MC approach. To facilitate this, we have provided a Python implementation of the model. Such model extensions have to be adjusted to the particular applications and the choice of the forcefield. Possible applications include the study of evaporation phenomena in complex systems, for example, liquid droplets with any kind of molecules, nanoparticles, etc. under different conditions (e.g., external fields). By using the framework of this study, these systems can be modeled by using any force-field (e.g., all-atom [34] and coarse-grained [35,36] models) at the particular temperature that the forcefield was obtained. Hence, our approach provides new areas of application for popular force-fields in nucleation and evaporation phenomena without the need for changing their parameters, since the processes are controlled at the LV interface by the chemical potential. The approach is flexible and can also be extended for more complex system setups suitable for studying bubbles or heat transfer processes between the substrate and the liquid and the vapor. In all these cases, different Monte-Carlo schemes can be used for generating the configurations and guaranteeing that certain criteria are met. We expect that in these cases the grid approach will be able to track the boundary between the liquid and the vapor phases, which is the crucial element for the success of our approach. Thus, we anticipate that our method will open new opportunities for the molecular-level simulation of nucleation and evaporation phenomena.

## Data Availability

The implementation of the method as a Python3.8 program is provided in the Supplementary Materials.

## Supplementary Materials

The following are available online at https://www.mdpi.com/article/10.3390/ma14092092/s1, Implementation of the method in Python3.8 Programming language including an example of an input file with associated documentation and a script to visualise the trajectories in povray. A movie demonstrating the dynamic equilibrium between the liquid droplet and the surrounding vapour. In this case, $T=\varepsilon /k_{B}$, μ=−0.4ε, $\varepsilon_{s}=1.0\varepsilon$, $r_{n}=1.5\sigma$, and $\rho_{c}=0.7\sigma^{-3}$.
